# Association of Hospital Matrons

**Published:** 1920-11-20

**Authors:** 


### Association of Hospital Matrons.

The fifth quarterly meeting of the Association of Hospital Matrons was held on Saturday last, by kind permission of the Treasurer and Almoners, in the Great Hall of St. Bartholomew's Hospital.

About 130 members were present—a very representative gathering from all parts of the country. The five local groups—namely, Yorkshire, East Lancs, Devon, and Cornwall, the Midlands and Winchester, had each sent their own delegate, and these ladies were warmly welcomed by Miss Lloyd Still, Matron of St. Thomas's Hospital, who presided over the meeting.

A most interesting paper on "The Nursing Profession in its Co-operation with Social Welfare Activities," was read by Miss A. E. Cummins, Lady Almoner, St. Thomas's Hospital, and a very full discussion took place on the subject of health work, and how the education of the trained woman could be expanded better to fit her for her duties of citizenship in this connection.

The inclusion of nurses under the Unemployment Insurance Act, and the possibility of hospitals for special diseases of women in affiliation with general hospitals being recognised as qualifying for the State Register under the General Nursing Council, were both questions brought forward and fully discussed.

At the close of the meeting Miss McIntosh entertained all present to tea, and the beauties of the Great Hall, with its fine paintings, were much appreciated by those of the members who had not had the opportunity of visiting it before. The membership of the Association now stands at 406.

